# Herbal therapy associated with antibiotic therapy: potentiation of the antibiotic activity against methicillin – resistant *Staphylococcus aureus *by *Turnera ulmifolia *L

**DOI:** 10.1186/1472-6882-9-13

**Published:** 2009-05-08

**Authors:** Henrique DM Coutinho, José GM Costa, Edeltrudes O Lima, Vivyanne S Falcão-Silva, José P Siqueira

**Affiliations:** 1 Laboratory of Research in Natural Products, Center of Biological Sciences and Health, University of the Region of Cariri, Crato (CE), Brazil; 2 Leão Sampaio College, Juazeiro do Norte (CE), Brazil; 3 Laboratory of Mycology, Center of Health Sciences, Federal University of Paraíba, João Pessoa (PB), Brazil; 4 Laboratory of Genetics of Microrganisms, Center of Exacts and Natural Sciences, Federal University of Paraíba, João Pessoa (PB), Brazil; 5 Universidade Regional do Cariri – URCA; Centro de Ciências Biológicas e da Saúde – CCBS; Departamento de Ciências Biológicas – DCB; Laboratório de Pesquisa em Produtos Naturais – LPPN. CEP: 63105-900. Crato, CE – Brasil. Rua Cel. Antonio Luis 1161, Pimenta, 63105-000

## Abstract

**Background:**

*Staphylococcus *genus is widely spread in nature being part of the indigenous microbiota of skin and mucosa of animal and birds. Some *Staphylococcus *species are frequently recognized as etiological agents of many animal and human opportunistic infections This is the first report testing the antibiotic resistance-modifying activity of *Turnera ulmifolia *against methicillin-resistant *Staphylococcus aureus *– MRSA strain.

**Methods:**

In this study an ethanol extract of *Turnera ulmifolia *L. and chlorpromazine were tested for their antimicrobial activity alone or in combination with aminoglycosides against an MRSA strain.

**Results:**

The synergism of the ethanol extract and aminoglycosides were verified using microdillution method. A synergistic effect of this extract on gentamicin and kanamycin was demonstrated. Similarly, a potentiating effect of chlorpromazine on kanamycin, gentamicin and neomycin, indicating the involvement of an efflux system in the resistance to these aminoglycosides.

**Conclusion:**

It is therefore suggested that extracts from *Turnera ulmifolia *could be used as a source of plant-derived natural products with resistance-modifying activity, constituting a new weapon against the problem of bacterial resistance to antibiotics demonstrated in MRSA strains.

## Background

*Staphylococcus *genus is widely spread in nature being part of the indigenous microbiota of skin and mucosa of animal and birds. Some *Staphylococcus *species are frequently recognized as etiological agents of many animal and human opportunistic infections [1]. *S. aureus, S. epidermidis, S. saprophyticus *and *S. haemolyticus *are the most important species as community and nosocomial human infection causing agents. In addition of causing different kinds of intoxications, *S. aureus *has been the most common etiological agent of festering infections that attack different tissues and/or organs (e.g. furuncle, carbuncle, abscess, myocarditis, endocarditis, pneumonia, meningitis, bacterial arthritis) [2,3]. Capsule, peptidoglican, teicoic acids, adesins and synthesis of enzymes and extracelullar toxins are some virulence attributes present in/on *S. aureus *cell [1].

Clinicians write millions prescriptions for antimicrobials every year [4]. Clinicians need to apply sound concepts when prescribing antimicrobials to both achieve a good outcome and avoid encouraging resistance. There is convincing evidence that inappropriate use of antibiotics directly leads to the development of resistant organisms [5]. To prevent this, is necessary educate all health worker regarding healthy drug use and regarding the natural history of the infection, emphasizing palliative therapies and infection control measures [6]. Infectious diseases are the major cause of morbidity and mortality and experience has shown that an approach that seeks to 'defeat' infectious diseases will not work. Long-term solutions must acknowledge this, and nurses and other health care professionals must take a proactive part in finding alternative solutions [7].

With increased incidence of resistance to antibiotics, natural products from plants could be interesting alternatives [8,9]. Some plant extracts and phytochemicals are known to have antimicrobial properties, and can be of great significance in therapeutic treatments. In the last few years, a number of studies have been conducted in different countries to demonstrate such efficacy [10-12].

Many plants have been evaluated not only for direct antimicrobial activity, but also as a resistance-modifying agent [13]. Several chemical compounds, synthetic or from natural sources, such as the phenothiazines, and natural products, have direct activity against many species of bacteria, enhancing the activity of a specific antibiotic, reversing the natural resistance of specific bacteria to given antibiotics, promoting the elimination of plasmids from bacteria and inhibiting transport functions of the plasma membrane in regard to given antibiotics. The inhibition of plasma membrane-based efflux pumps has been observed as well [14,15]. The enhancement of antibiotic activity or the reversal of antibiotic resistance by natural or synthetic non-conventional antibiotics affords the classification of these compounds as modifiers of antibiotic activity.

*Turnera ulmifolia *L. (Turneraceae), a small annual herb, can be found in the north and northeast brazilian regions, where it is considered a weed [16]. It grows preferentially in sandy soils and on hill slopes. *T. ulmifolia *L. is already known to be of medicinal value, being used popularly as an anti-inflammatory, as an expectorant, and in the treatment of several problems [16-18]. Authors detected flavonoids, alkaloids, tannins and phenolic compounds in preparations from this plant [19-21].

Aminoglycosides are potent bactericidal antibiotics targeting the bacterial ribosome, and the increase in cases of bacterial resistance to aminoglycosides is widely recognized as a serious health threat [22]. The main mechanisms of resistance to aminoglycosides are active efflux and enzymatic inactivation [23].

In this work, we tested an ethanol extract of *Turnera ulmifolia *as a resistance modifying agent in an aminoglycoside-resistant strain of *S. aureus*.

## Methods

### Strains

The strain used was the clinical isolate *Staphylococcus aureus *358 (SA358), resistant to several aminoglycosides [24]. The SA-ATCC25923 strain of *Staphylococcus aureus *was used as a positive control. The strains were maintained in heart infusion agar slants (HIA, Difco), and prior to assay, the cells were grown overnight at 37°C in brain heart infusion (BHI, Difco).

### Plant material

Leaves of *Turnera ulmifolia *were collected in the county of Crato, Ceará State, Brazil. The plant material was identified and a voucher specimen was deposited with the number 1618 at the Herbarium "Dárdano de Andrade Lima" of Universidade Regional do Cariri – URCA.

### Preparation of ethanol extract of Turnera ulmifolia (EETU)

A quantity of 200 g of leaves were dried at room temperature and powdered. The powdered material was extracted by maceration using 1 L of 95% ethanol as solvent at room temperature, and the homogenate was allowed to stand for 72 h at room temperature. The extracts were then filtered and concentrated under vacuum in a rotary evaporator [25]. For the tests, the dry extract material was dissolved in DMSO (dimethyl sulfoxide) 0,5%. The DMSO was chosen due its less toxicity than ethanol.

### Drugs

Chlorpromazine, gentamicin, tobramycin, kanamycin, amikacin and neomycin were obtained from Sigma Chemical Co. All drugs were dissolved in sterile water.

### Drug susceptibility test

The minimum inhibitory concentration (MIC) of EETU, antibiotics and chlorpromazine (CPZ) were determined in BHI by the microdilution assay using suspensions of 10^5 ^CFU/ml and a drug concentration range of 1024 to 1 μg/ml (twofold serial dilutions) [26]. MIC was defined as the lowest concentration at which no growth was observed. For the evaluation of EETU as a modulator of antibiotic resistance, MICs of the antibiotics were determined in the presence of EETU (32 μg/ml) and CPZ (16 μg/ml) at sub-inhibitory concentrations, and the plates were incubated for 24 h at 37°C. CPZ was used as positive control for efflux pump inhibition [27]. Each experiment was undertaken in duplicate.

## Results

The EETU did not show a substantial antibacterial activity at 1024 μg/ml against the strains assayed (MIC ≥ 1024 μg/ml). None effect was observed when EETU was combined with the aminoglycosides in the strain SA-ATCC25923.

The addiction of EETU to the growth medium at 32 μg/ml produced a dramatic reduction in the MIC for gentamicin and kanamycin in the strain *Staphylococcus aureus *358, demonstrating a potentiating effect of EETU on aminoglycoside activity (Table 1).

**Table 1:** MIC‡ values (μg/ml) of aminoglycosides in the absence and presence of EETU# and CPZ * in *Staphylococcus aureus 358*.

	SA358		
	MIC	MIC combined	

Antibiotics		EETU (32 μg/ml)	CPZ (16 μg/ml)
Amikacin	8	8	8
Gentamicin	8	<1	4
Kanamycin	≥ 1024	128	8
Neomycin	16	16	8
Tobramycin	8	8	8
EETU	≥ 1024	-	-

Chlorpromazine	16	-	-

‡MIC – Minimal Inhibitory Concentration;.*# *EETU – Ethanolic Extract of *Turnera ulmifolia*; * CPZ – chlorpromazine

A MIC reduction for gentamicin and kanamycin was also observed when CPZ was added to the growth medium at 16 μg/ml, which indicates the involvement of an efflux pump in the resistance to these antibiotics (Table 1).

A potentiating effect of CPZ on amikacin and tobramycin was not observed, which suggests the occurrence of other resistance mechanisms (Table 1).

## Discussion

Only few articles were published focusing pharmacological activities of the genus *Turnera*. Some species of *Turnera *are widely used in folk medicine for different types of inflammatory diseases. Fresh leaves of *T*. *guaianensis *Aubl. is used to treat inflammatory diseases in general and as an immunomodulator while the decoction of it's dried leaves is employed to treat furunculosis [17]. *T*. *diffusa *Willd., in a decoction of the whole plant, is used to treat otitis and nephritis [28,29], while the leaf infusion is used in diseases related to the gastrointestinal and respiratory systems [30], reproductive organs in general or specifically for gonorrhoea treatment [17]. *T. ulmifolia *is used popularly as an anti-inflammatory and as an expectorant [16-18], but as far as we know, natural products of *Turnera ulmifolia *or of any plants from the genus *Turnera *having a potentiating effect on any drug, mainly antibiotics (as aminoglycosides) have not been previously reported in human or animals.

Phenothiazines, such as chlorpromazine, probably act on the plasma membrane of bacteria affecting the efflux pumps [31]. This modification of permeability could enhance the activity of antibiotics that act within the cell, such as the aminoglycosides.

Efflux pumps have been associated with resistance mechanisms since the 1980s^32^, representing one of the main mechanism of multidrug resistance (MDR) evolved in the antibiotic resistance to aminoglycosides [32]. Several studies have been performed to identify drugs interfering with these pumps, called resistance modifying agents [33]. Other plant products, as ethanol extract of *Mentha arvensis*, affected the efflux system of an *Escherichia coli *multiresistant to aminoglycosides, inhibitinh these resistance mechanism [27].

This strategy is named "herbal shotgun" or "Synergistic multi-target effects" and refers to the use of herbals and drugs in a multitargeted approach, due the fact of mono or multi-extract combinations affect not only a single target, but several ones, cooperating in an agonistic-synergistic way. This approach are not exclusive for extract combinations, but combinations between single natural products or extracts with chemosynthetics or antibiotics are possible too [34,35].

## Conclusion

The results obtained indicate that *Turnera ulmifolia *(and broadly Turneraceae) could serve as a source of plant-derived natural products with antibiotic resistance-modifying activity to be used against multiresistant bacteria as MRSA strains acquired from hospital and community.

## Competing interests

The authors declare that they have no competing interests.

## Authors' contributions

HDMC was the principal investigator, participated in the planning and execution of the study, performed data entry and data analysis, and was the main responsible author. JGMC, EOL, VSFS and JPSJ participated in the planning of the study and contributed to the writing process.

## Pre-publication history

The pre-publication history for this paper can be accessed here:


